# Stakeholders’ opinions and questions regarding the anticipated malaria vaccine in Tanzania

**DOI:** 10.1186/s12936-016-1209-6

**Published:** 2016-04-05

**Authors:** Sally Mtenga, Angela Kimweri, Idda Romore, Ali Ali, Amon Exavery, Elisa Sicuri, Marcel Tanner, Salim Abdulla, John Lusingu, Shubi Kafuruki

**Affiliations:** Ifakara Health Institute (IHI), P.O. Box 78373, Dar es Salaam, Tanzania; ISGlobal, Barcelona Centre International Health Research (CRESIB), Hospital Clínic, Universitat de Barcelona, Barcelona, Spain; Health Economics Group, Department of Infectious Disease Epidemiology, School of Public Health, Imperial College London, London, UK; National Institute for Medical Research Institute (NIMR), Tanga, Tanzania; Swiss Tropical and Public Health Institute, Basel, Switzerland

**Keywords:** Stakeholders, Opinions, Malaria vaccine, Tanzania, Questions

## Abstract

**Background:**

Within the context of combined interventions, malaria vaccine may provide additional value in malaria prevention. Stakeholders’ perspectives are thus critical for informed recommendation of the vaccine in Tanzania. This paper presents the views of stakeholders with regards to malaria vaccine in 12 Tanzanian districts.

**Methods:**

Quantitative and qualitative methods were employed. A structured questionnaire was administered to 2123 mothers of under five children. Forty-six in-depth interviews and 12 focus group discussions were conducted with teachers, religious leaders, community health workers, health care professionals, and scientists. Quantitative data analysis involved frequency distributions and cross tabulations using Chi square test to determine the association between malaria vaccine acceptability and independent variables. Qualitative data were analysed thematically.

**Results:**

Overall, 84.2 % of the mothers had perfect acceptance of malaria vaccine. Acceptance varied significantly according to religion, occupation, tribe and region (p < 0.001). Ninety two percent reported that they will accept the malaria vaccine despite the need to continue using insecticide-treated nets (ITNs), while 88.4 % reported that they will accept malaria vaccine even if their children get malaria less often than non-vaccinated children. Qualitative results revealed that the positive opinions towards malaria vaccine were due to a need for additional malaria prevention strategies and expectations that the vaccine will reduce visits to the health facility, deaths, malaria episodes and treatment-related expenses. Vaccine related questions included its side effects, efficacy, protective duration, composition, interaction with other medications, provision schedule, availability to the pregnant women, mode of administration (oral or injection?) and whether a child born of HIV virus or with a chronic illness will be eligible for the vaccine?

**Conclusion:**

Stakeholders had high acceptance and positive opinions towards the combined use of the anticipated malaria vaccine and ITNs, and that their acceptance remains high even when the vaccine may not provide full protection, this is a crucial finding for malaria vaccine policy decisions in Tanzania. An inclusive communication strategy should be designed to address the stakeholders’ questions through a process that should engage and be implemented by communities and health care professionals. Social cultural aspects associated with vaccine acceptance should be integrated in the communication strategy.

## Background

Malaria remains a major public health concern in sub-Saharan Africa (SSA). Tanzania is one of the countries in which malaria continues to be a significant cause of morbidity, mortality and considered as an impediment to social economic growth and welfare [1]. According to the National Malaria Control Programme (NMCP), 90 % of the Tanzanian population are at risk of malaria, resulting into 11 million clinical cases per year. The most vulnerable to malaria are children and pregnant mothers [2]. In Tanzanian mainland, the number of malaria microscopically-confirmed cases are 1,550,250 and reported deaths are 8525 [3]. Despite a declining trend in the number of admissions and deaths over the last few years, the country experiences a marked variation across regions having some with high malaria prevalence and others with low prevalence. For instance, there are regions with one percent or less and others with more than 30 % [4]. The current malaria interventions in Tanzania include malaria testing by microscopy and/or rapid diagnostic tests, treatment with affordable and effective malaria treatment, such as artemisinin-based combination therapy (ACT), protection using long-lasting insecticide-treated nets and indoor residual spraying with insecticides, intermittent preventive therapy with sulfadoxine-pyrimethamine for pregnant women [2]. However, challenges are also reported on existing malaria interventions with regards to resistance of malaria parasites to ACT, as well as non-use of mosquito nets [5, 6]. Considering variations in malaria prevalence and challenges related to the existing malaria interventions, more innovative response including the vaccines to prevent malaria is likely to improve the impact of available interventions.

Vaccines are considered effective interventions in protecting individuals from infectious diseases and the best tool to achieving disease eradication in various contexts [7]. The currently most advanced candidate vaccine RTS, S/AS01 against *Plasmodium falciparum* malaria, has been tested across several sub-Saharan African countries including Tanzania. Phase 3 trials showed that during 12 months of follow-up, half malaria episodes were protected in 5–17 months. One third malaria episodes were protected in 6–12 weeks cohort [8]. In infants 6–12 weeks of age, vaccine efficacy was about 30 % against both for clinical and severe malaria [9]. Recent study indicates that during 18 months of follow up, vaccination of children and young infants with RTS, S/AS01 prevented many cases of clinical and severe malaria and that the vaccination showed the highest impact in regions with the highest incidence of malaria [10]. Tanzania with other countries in Africa is underway to launch a malaria vaccine which is hoped to cut episodes of clinical malaria in young children by about half [11]. In the context of the current efficacy results a policy recommendation is likely to occur paving a way for the implementation of the vaccine in countries through their expanded programmes on immunization.

Although stakeholders’ (community and professionals) voice is imperative before policy endorsement [12]; to date, there is limited information regarding their acceptance and questions related to the vaccine. Where information on the acceptance of the malaria vaccine exists [13, 14], it is not incorporated within the context of the ongoing malaria interventions and does not highlight on whether people may be willing to undertake the vaccine even when it is unlikely to provide full protection. Moreover, the accounts of contextual aspects that influence vaccine acceptance are not fully presented. Such information is crucial for policy decisions and future implementation if recommendation on the vaccine is made in the near future. However such information is missing in Tanzania despite being one of the country in which the RTS, S/AS01 vaccine trial was implemented. Experience indicates that it takes time for the interventions to gain public acceptance even after it has been licensed due to various factors including community acceptance and inadequate prior information that could inform the policy makers on what need to be considered before the implementation of the intervention [14]. Also, the absence of critical data could slow down the decision process that policymakers must undertake to determine whether or not to introduce a particular intervention into their health systems [15]. In addition, lack of community support due to poor knowledge and perceptions made community delay the uptake while others reject vaccines. For instance, it existed when Polio vaccination programme was delayed in northern Nigeria [16]. Therefore, it is crucial that community perceptions are understood and used to highlight any community-based issues that need to be considered during policy deliberation and intervention planning [17].

Within the context of planning for a vaccine to be used alongside existing malaria control methods, mothers of children under five and other stakeholders (teachers, religious leaders, community health workers, health care professionals and scientists) were interviewed to assess their perceptions on malaria and acceptance of the malaria vaccine.

The following were the specific objectives:

- To determine stakeholders’ acceptance of the anticipated malaria vaccine and the associated factors.
- To assess stakeholders’ perception and attitude towards the vaccines.
- To explore stakeholders’ expectations from the anticipated malaria vaccine.
- To explore stakeholders’ questions with regards to the anticipated malaria vaccine.

We hope that the study findings may assist the policy makers in Tanzania to make informed decisions on the introduction of malaria vaccine in line with other existing malaria intervention strategies [15]. The data may also inform the design of the communication strategy and guide the country’s programmers on the issues to be considered before the actual implementation of the vaccine.

## Methods

### Overall study design

A cross sectional study that involved quantitative and qualitative methods was conducted. The study was implemented between May and June 2013 in twelve districts of Tanzanian mainland (Table 1). The mixed method approach aimed at triangulating the methods and findings for completeness of the data [18]. The participating districts were from areas where phase II and III RTS, S malaria vaccine trial had not been implemented. This was done purposely to minimize bias of opinion with regards to acceptance of malaria vaccine. The districts were from the northern, eastern, western, central and southern parts of the country for enhancing the representativeness of voices from communities of diverse background since the future malaria vaccine may not only be introduced in trial sites. Malaria in the study regions ranges from 1 % in Arusha to more than 20 % in Lindi and Mtwara [1]. In the country, EPI coverage is high but varies across regions having the highest coverage in Arusha (100 %) and the lowest coverage in Kagera (57 %) [19]. The health care system in Tanzania is composed of the public hospitals and the private hospitals. Hospitals are the highest level of access to care and the dispensary being the lowest level. However, at the dispensaries is where the majority of people in urban and rural communities access their health care.

**Table 1:** Study regions and the selected districts

Regions	Districts
Arusha	Ngorongoro
Tanga	Handeni
Kagera	Ngara
Pwani	Kisarawe
Mtwara	Newala
Mbeya	Ileje
Mara	Serengeti
Lindi	Lindi rural
Mwanza	Ilemela
Morogoro	Morogoro Municipal/Kilombero
Dar es Salaam	Kinondoni
Dodoma	Dodoma Municipal

### Qualitative methods

#### Design and setting

Using qualitative approach, the study employed parallel individual interviews (IDIs) and focus groups discussions (FGDs). Qualitative participants were from some of the study sites where the quantitative study was conducted including participants from Mwanza (Ilemela district), Mbeya (Ileje district) and Arusha (Ngorongoro district). Addition participants were from Morogoro and Dar es Salaam regions. Preference to conduct qualitative study in these sites was based on the convenience and cost.

### Study population and recruitment

Forty six IDIs and 12 FGDs were conducted. The IDI participants comprised of primary school teachers, religious leaders, community health workers, health care professionals, and scientists. The scientists who participated in the discussion were from various professional backgrounds (sociologists, medical doctors, public health, epidemiologists) excluding those who were participating in the clinical trials. FGDs were carried on with men and women in the respective study sites. The FGDs allowed insights into general group norms on the vaccines and capturing varied views and questions with regards to the anticipated malaria vaccine. The IDIs involved individuals who were believed to be capable of providing personal opinions about malaria and the anticipated malaria vaccine. The participants in the local communities were purposively [20] selected by the assistance of the community leaders believed to be influential in decisions about health-seeking practice in their families and community at large. Scientists were recruited from various institutions both public and private. Selection of the scientists was mostly based on the convenience and availability of the individuals in their institutions. The health care professionals were recruited based on their assimilation with child care services i.e. working in the reproductive health unit and paediatric care.

### Data collection

Focus group guide with open ended questions was used to collect FGD data, while a semi structured topic list was used to collect IDI data. Both tools addressed similar topics directly designed to address specific study objectives. The tools were adjusted according to the best fit of the study audience. Main topics included: perception about malaria status, perception about malaria vaccine, expectations from malaria vaccines, preferred modality of providing malaria vaccine and questions with regards to malaria vaccine. IDI and FGD tools were piloted for practicability. Experienced research assistants conducted the IDIs and FGDs. Prior to data collection experienced social scientists (SM, AK) provided training to the research assistants. The training familiarized them with the study objectives, status of malaria vaccine research, interview procedures and ethical aspects. The FGDs were made of up to 7–9 participants and composed of a moderator who facilitated the discussion and a note taker who assisted in taking notes. Both discussions and interviews were conducted in a place convenient to the study participants. To enhance freedom of discussion, women and men had separate discussion groups. The discussion sessions were conducted in Kiswahili, a Tanzania national language which is well understood and used commonly in the study area. Subject to participants’ consent some data from interviews with communities were audio-taped but other data was put in the expanded notes [21]. The interviews and discussions lasted for 1 h and 30 min on average.

### Data analysis

Audio-recorded data was transcribed verbatim for analysis. The transcripts and expanded notes were checked for completeness and accuracy. Two social scientists (SA and AK) experienced in qualitative studies independently reviewed the transcripts to identify the relevant patterns and later the patterns were grouped into main themes. N-VIVO program [22] assisted in the display of participants expressions and the coding process. Considering the views of various stakeholders, a constant comparison approach was employed to compare themes that emerged from these analytic procedures [23]. To consolidate results, the identified themes and categories were shared in a malaria vaccine working groups and during the national stakeholders’ meeting.

The stakeholders from immunization department in Tanzania and others discussed the findings and the consensus was reached about the interpretation of the results. All data were analysed in the Kiswahili but relevant quotes were translated into English for the purpose of this paper.

### Quantitative methods

The study involved face to face household interviews with women aged 18 years and above, who had at least one child under 5 years. Quantitative study was employed to estimate level of acceptance of the malaria vaccine and preferred modality of providing malaria vaccine. The structured questionnaire was used to interview the eligible mothers. Before the interviews, the tools were piloted and later necessary changes were adopted in the tools.

A multi-stage random sampling was used to select the households in the specific study areas. At first the country was stratified based on regions, one region was randomly selected from which a list of districts was sought and then one district was randomly selected from the list of districts, (excluding the two districts that have been involved in malaria vaccine trial that is Bagamoyo and Korogwe), followed by village, and lastly household. The sample size was calculated based on simple random sampling given by the following formula and values attributed to parameters:

$$ n = \frac{{Z^{2} p(1 - p)N}}{{d^{{2(N - 1) + Z^{2} p(1 - p)}} }} $$

n = sample size; z–score, which is the number of standard deviations from the mean. At 95 % confidence level, z = 1.96; p = prevalence of malaria (assumed to be 50 %); d = absolute precision required (1 %); N = population size (different according to the region, ranging from 188 to 332).

Thereafter, the sample size from simple random sample was multiplied by design effect to take into account the clustering effect. The design effect is given by:

$$Def = 1 + \left({Average\;population\;per\;cluster - 1} \right) \times \rho$$

where, ρ is a cluster correlation. The sample size was required to detect 50 % proportion on perception of malaria vaccine. The sample size calculation was to give idea of how many individuals were deemed to be interviewed from each study district. Prior to data collection, the research team received orientation on the purpose of the study, reminded about ethical aspects and review of the questions. Electronic devises (tablets) were used to collect information during the interviews, and later the information was synchronized into the field computers. Quality check and errors were carried out in the field and prompt feedback was provided to the field supervisors and later to the research assistants for corrective measures.

All the quantitative data were managed and analysed using STATA 11 Software (Stata corp, USA). Data analysis was performed as both one-way frequency distributions and cross tabulations of various outcomes against selected independent variables. In the latter case, Chi Square (χ^2^) was used to test the degree of association between each pair of categorical variables involved in a cross-tabulation. The significance was determined at p ≤ 0.05. Acceptability of the anticipated malaria vaccine was the main variable and was defined by three questions;

(A) Researchers are working to develop a malaria vaccine, which will have the possibility of reducing the recurrence of malaria among children. If the malaria vaccine becomes available will you be willing for your child to receive that malaria vaccine?
(B) Malaria vaccine may cause discomfort similar to other childhood vaccines will you agree or disagree that your child still get vaccinated?
(C) Even though a child is vaccinated, s/he will still have to use ITNs and seek treatment if s/he has fever. Will you agree that your child get vaccinated?

Frequency distribution of responses in each of these questions (A, B and C) was performed.

Then bivariate analysis of each question, independent of the other, was conducted to assess how each of these outcomes was related to background and non-background characteristics of the participants, such as age, education, religion and region. Finally, these variables were combined to form a single powerful indicator of acceptability, such that:

$${\rm Acceptance} \,\,{\rm of} \,\,{\rm  malaria} \,\,{\rm vaccine} =  \left\{ \begin{array}{ll} {\text{PERFECT}} &\quad {\text{if A }} = {\text{ YES and B }} = {\text{ YES and C }} = {\text{ YES}}  \\ {\text{PARTIAL}} &\quad {\text{if YES to any one or two but not all of the A}},{\text{ B and C}}  \\ {\text{NO}}&\quad  {\text{if A }} \ne {\text{ YES}}   \end{array} \right. $$

### Ethical aspects

The study was approved by the ethical review board of the Ifakara Health Institute (IHI-IRB). Prior to interviews, local authorities were contacted and asked for permission. Written consent to approach the study communities was obtained. Verbal and written informed consents were obtained from all study participants through which participants were assured of anonymity and confidentiality of information.

## Results

Since the study was a mixed method design, results are triangulated for the purpose of elaboration and completeness. Results are presented in general themes emanated from the study objectives. Qualitative findings are not presented according to study groups due to observed convergence of views and opinions with regards study phenomenon.

### Characteristics of study respondents

Out of a sample size of 2124, a total of 2123 mothers with children under five from nine districts participated in the study. Of the 2123 mothers, 70 % were in the age range of 20 and 34 years. A majority of mothers (84 %) were in marital relations. Slightly more than one third (34.7 %) of the participants had more than three children. The study population was relatively literate with only 19.4 % of respondents who had never attended school, whilst about 69.4 % had attained primary school and 11 % had secondary education. A majority (70.4 %) of respondents were farmers. About 56.2 % of respondents were Christians and 40.9 % were Muslims (Table 2).

**Table 2:** Characteristics of the respondents in 2013 household survey (n = 2123)

Characteristics	Number of respondents (n)	Percent (%)
Overall	2123	100.0
Age (years)		
<20	143	6.7
20–34	1499	70.6
>34	481	22.7
Mean = 28.9 ± 7.6, min = 15.0, max = 65.0	–	–
Marital status		
Currently married	1793	84.5
Ever married (currently divorced/widowed)	143	6.7
Single	187	8.8
Education		
Never been to school	412	19.4
Primary	1473	69.4
Secondary+	238	11.2
Parity (number of children)		
1	537	25.3
2	473	22.3
3	376	17.7
4+	737	34.7
Occupation		
Farmer/other	1495	70.4
Business (petty vender, tailoring etc)	355	16.7
Housekeeper/no job	215	10.1
Civil servant	58	2.7
Religion		
Islam	868	40.9
Christian	1192	56.2
Other	63	3.0
Tribe		
Kurya	313	14.7
Makonde	272	12.8
Hangaza	206	9.7
Zigua	204	9.6
Zaramo	140	6.6
Ndali	129	6.1
Sukuma	120	5.7
Others^a^	739	34.8
Region (district)		
Arusha (Ngorongoro)	280	13.2
Kagera (Ngara)	331	15.6
Lindi (Lindi rural)	183	8.6
Mara (Serengeti)	286	13.5
Mbeya (Ileje)	188	8.9
Mtwara (Newala)	193	9.1
Mwanza (Ilemela)	235	11.1
Pwani (Kisarawe)	190	9.0
Tanga (Handeni)	237	11.2

^a^ Sonjo, Mwela, Yao, Iraki, Chagga, Ha, Masai, Haya, Zigua, Sambaa etc

Qualitative participants composed of 21 health care professionals (18 health care providers and four paediatricians), six teachers, four religious leaders and six community health workers. Twelve more IDIs were conducted with scientists from various institutions in Dar es Salaam (Table 3). FGDs were carried on with six groups of women and six groups of men. Most of the FGD and IDI participants were of age 25 and 50. Non-professional participants were mostly farmers and petty trade dealers. Most of them had attained primary school level. The majority of the professionals, such as nurses and teachers, were of secondary school and high school levels.

**Table 3:** Summary of the IDIs and FGDs participants

	Further details	Total number of participants
IDIs		
Health care professionals		
Nurses	From public health facilities and private health facilities	12
Paediatricians	From public health facilities	3
Paediatricians	From private health facilities	3
Teachers	From rural community	6
Religious leaders	From Muslim community	2
Religious leaders	From Christian community	2
Community health workers	From rural community	6
Scientists	From sociological background	3
Scientists	From medical background	3
Elites	From government and private institutions	6
Total		45
FGDs		
Women	From rural community	6
Men	From rural community	6
Total		12

### Perception and attitude towards vaccines

Qualitative participants (mostly women) possessed a positive opinion towards vaccines. They were in the opinion that the vaccines are important for the reduction of disease severity, reduced cost of treatment and disease prevention.

One female participant expressed her opinion that vaccine would reduce the severity of disease:

*“I know that when a child gets vaccinated he will be protected from diseases. Even if the disease comes, it will not be very much severe as compared to if the child has not completely received a vaccine”* (FGD, Female_04).

Another female participant was in the opinion that vaccine is important for prevention of diseases:

*“Just like what the experts says “it is better to prevent than to cure” then I think vaccination is important as it helps to prevent a child from diseases and reduces treatment costs because during treatment you use much cost to treat the child unlike when the child is protected (with the vaccine)”* (FGD, Female_05).

**Fig. 1:**
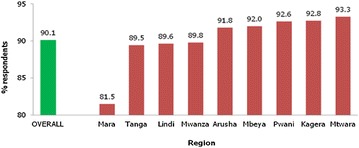
Percent distribution of respondents that believe that there are benefits related to under-five child vaccination (n = 2123)

Similarly in the quantitative study, the majority (90.1 %) of mothers reported that there is a benefit associated with vaccination (Fig. 1). Also, about 97.6 % agreed with the statement that ‘I prefer my child to receive all the vaccines’ (Table 4).

**Table 4:** Perception towards vaccine: percent distribution of respondents that AGREE with the listed statements by region (n = 2123)

	Number of respondents	% Stated that …		
		I prefer my child to have all vaccinations	I prefer my child to have certain vaccinations	I prefer my child not to be vaccinated at all
Overall	2123	97.6	0.7	0.1
Region				
Arusha	280	98.2	1.1	0.4
Kagera	331	98.8	0.0	0.0
Lindi	183	97.3	1.6	0.0
Mara	286	99.0	1.4	0.0
Mbeya	188	100.0	0.0	0.0
Mtwara	193	97.9	0.0	0.0
Mwanza	235	98.7	0.9	0.0
Pwani	190	91.6	0.0	0.0
Tanga	237	94.9	0.8	0.0

### Acceptance of malaria vaccine and the associated reasons

Most of the opinions of the qualitative participants reflected a positive acceptance towards the anticipated malaria vaccine. The main consensus was that malaria vaccine is important since malaria is still a common disease among children under five.

One of the paediatricians provided his view that malaria vaccine need to be provided since more strategies are needed to fight malaria:

*“I think malaria problem is still there and more weapons are needed in making sure that it is prevented, vaccine is one of the weapon, but if it’s safe for the users”* (IDI, Paediatrician _05).

Another participant was in the opinion that malaria still affects children and hence a need to introduce malaria vaccine:

*“Malaria vaccine should be introduced due to the burden of malaria especially for young children”* (IDI, Nurse, RCH_06).

A male participant thought that malaria vaccine is needed because the mosquito nets cannot provide full protection from mosquitoes:

*“I think we need malaria vaccine since we are not always covered by the mosquito nets. Look at where we are now, we have stayed for almost one hour and the mosquito nets are inside our houses on the beds. Probably the mosquitoes might have already bitten the child. Therefore, we cannot totally depend on the mosquito nets …”* (FGD, Male_ 05).

The quantitative results revealed that the majority (84.2 %) of the participants indicated a perfect acceptance of malaria vaccine, 11.9 % had partial acceptance while 3.9 % had no acceptance of the vaccine (Table 5). Occupation, tribe, religion, and regions attained a statistical significance with the perfect acceptance of the malaria vaccine (p < 0.001), with farmers, Christians, members of the tribe Hangaza and households in the Kagera region presenting higher acceptance levels.

**Table 5:** Degree of malaria vaccine acceptability by various characteristics (n = 2123)

Characteristics	Total number of respondents	Degree of acceptability (%)			P value
		Perfect acceptance	Partial acceptance	No acceptance	
Overall	2123	84.2	11.9	3.9	‒
Age (years)					
<20	143	83.9	14.0	2.1	0.466
20–34	1499	83.6	12.3	4.1	
>34	481	86.1	10.2	3.7	
Marital status					
Currently married	1793	84.5	11.4	4.1	0.149
Ever married (currently divorced/widowed)	143	79.7	18.2	2.1	
Single	187	84.5	11.8	3.7	
Education					
Never been to school	412	87.4	10.2	2.5	0.136
Primary	1473	83.9	12.0	4.1	
Secondary+	238	80.3	14.3	5.5	
Parity (number of children)					
1	537	81.8	14.3	3.9	<0.169
2	473	82.0	13.5	4.4	
3	376	85.6	10.1	4.3	
4+	737	86.6	10.0	3.4	
Occupation					
Farmer/other	1495	86.1	10.5	3.4	*<0.001*
Business (petty vender, tailoring etc)	355	81.1	14.7	4.2	
Housekeeper/no job	215	77.7	17.7	4.7	
Civil servant	58	77.6	10.3	12.1	
Religion					
Islam	868	79.7	13.5	6.8	*<0.001*
Christian	1192	87.3	10.8	1.9	
Other	63	87.3	11.1	1.6	
Tribe					
Kurya	313	87.5	10.9	1.6	*<0.001*
Makonde	272	79.8	16.5	3.7	
Hangaza	206	93.7	4.9	1.5	
Zigua	204	90.2	6.4	3.4	
Zaramo	140	76.4	10.0	13.6	
Ndali	129	90.7	8.5	0.8	
Sukuma	120	80.8	15.8	3.3	
Others^a^	739	80.9	14.5	4.6	
Region (district)					
Arusha (Ngorongoro)	280	80.0	16.8	3.2	*<0.001*
Kagera (Ngara)	331	93.4	5.1	1.5	
Lindi (Lindi rural)	183	78.1	16.4	5.5	
Mara (Serengeti)	286	86.7	11.2	2.1	
Mbeya (Ileje)	188	90.4	9.0	0.5	
Mtwara (Newala)	193	79.8	16.1	4.2	
Mwanza (Ilemela)	235	81.3	16.2	2.6	
Pwani (Kisarawe)	190	72.1	15.8	12.1	
Tanga (Handeni)	237	89.0	4.6	6.3	

^a^ Sonjo, Mwela, Yao, Iraki, Chagga, Ha, Masai, Haya, Sambaa etc

### Expectations from malaria vaccine

The common expectations from the malaria vaccine by most participants comprised a view that malaria vaccine will lessen the malaria episodes, frequent visits to the hospital due to malaria, the number of deaths and that the overall burden of malaria among children will be reduced.

*“My expectations is that if malaria vaccine will work, it will help reduce the hassle we get of having frequent malaria, you will find a child going back to hospital even four times in a month”* (FGD, Male _03).

*“The expectations of most people will be that the malaria vaccine will completely eradicate malaria, because the children will have protection…and so malaria will finish****…”*** (FGD, Female_05).

*“The health care providers will feel very proud to have this additional vaccine on top of the existing ones since we hope it will succeed in reducing the mortality rate especially for children under 5* *years”* (Nurse, RCH_05).

*“Most mothers will definitely take their kids for vaccination since the costs of treatment nowadays is very high”* (Teacher_02).

### Acceptance of malaria vaccine in the context of ITN use

The majority (92.5 %) reported that they will be ready to take their children for malaria vaccine despite their obligation to use ITNs and seek treatment when the child has fever. There were differences in the level of acceptance across regions, religion and tribe. The Mbeya region was more likely to indicate acceptance comparatively to other study regions (p = 0001) (Table 6).

**Table 6:** Percent distribution of respondents ready to get their children vaccinated with malaria vaccine despite the fact that even though a child is vaccinated, s/he will still have to use ITNs and seek treatment if s/he has fever; by various characteristics (n = 2123)

Characteristics	Total number of respondents	% Ready	P value
Overall	2123	92.5	‒
Age (years)			
<20	143	93.7	0.798
20–34	1499	92.3	
>34	481	92.7	
Marital status			
Currently married	1793	92.5	0.742
Ever married (currently divorced/widowed)	143	90.9	
Single	187	93.1	
Education			
Never been to school	412	92.5	0.962
Primary	1473	92.5	
Secondary+	238	92.0	
Parity (number of children)			
1	537	93.1	0.446
2	473	90.9	
3	376	92.0	
4+	737	93.2	
Occupation			
Farmer/other	1495	93.1	0.125
Business (petty vender, tailoring etc)	355	90.7	
Housekeeper/no job	215	92.6	
Civil servant	58	86.2	
Religion			
Islam	868	88.5	*<0.001*
Christian	1192	95.1	
Other	63	96.8	
Tribe			
Kurya	313	94.6	*<0.001*
Makonde	272	91.2	
Hangaza	206	96.1	
Zigua	204	94.1	
Zaramo	140	82.1	
Ndali	129	99.2	
Sukuma	120	92.5	
Others^a^	739	91.3	
Region (district)			
Arusha (Ngorongoro)	280	92.1	*<0.001*
Kagera (Ngara)	331	96.7	
Lindi (Lindi rural)	183	88.5	
Mara (Serengeti)	286	93.7	
Mbeya (Ileje)	188	99.5	
Mtwara (Newala)	193	90.7	
Mwanza (Ilemela)	235	93.6	
Pwani (Kisarawe)	190	81.1	
Tanga (Handeni)	237	92.4	

^a^ Sonjo, Mwela, Yao, Iraki, Chagga, Ha, Masai, Haya, Sambaa etc

### Acceptance of malaria vaccine in the context of partial protection

Participants were asked to provide their views on how they think about accepting the forthcoming malaria vaccine despite that their children will get malaria less often than those who don’t get the vaccine. Most participants views consistently indicated a willingness to uptake malaria vaccine in the context of its partial protection due to their concern with the burden of malaria and the view that the less the episodes the less the cost of treatment.

*“This malaria vaccine need to be introduced because it will reduce the magnitude of malaria, even if it reduces to some extent, it is still important, because if a child gets malaria less frequently different from now, the costs of treatment will reduce…” (IDI, Religious leader_02).*

*“If efficacy is 50* % *is fine as long as you have helped the person by reducing the episodes. This will help to enhance immunity”* (IDI, Health professional lecturer_04).

*“Vaccine would be the best solution, even if it is partially protected then should be introduced. It should go parallel with other strategies to be more effective.”* (IDI_Paediatrician_3).

“…. *even with existing vaccines, still the children get sick but not as much as those who did not get vaccine at all”* (Teacher_03).

The views of the qualitative participants converged with the quantitative data which indicated that the majority of mothers (88.4 %) reported that they will be comfortable that their children receive malaria vaccine despite that they will still get malaria less often than those who don’t get the vaccine. Age of mothers, religion, region and tribes were statistically significantly associated with the acceptance of partial protection of malaria vaccine (Table 7).

**Table 7:** Percent distribution of respondents that answered “YES” to the question “If your child receives malaria vaccine, and still gets malaria but less often than those who don’t get vaccine, will you be comfortable with that?”; by various characteristics (n = 2123)

Characteristics	Total number_of respondents	% That would be comfortable	P value
Overall	2123	88.4	–
Age (years)			
<20	143	83.2	*0.010*
20-34	1499	87.9	
≥34	481	91.7	
Marital status			
Currently married	1793	88.7	0.559
Ever married (currently divorced/widowed)	143	86.7	
Single	187	86.6	
Education			
Never been to school	412	87.9	0.863
Primary	1473	88.7	
Secondary+	238	87.8	
Parity (number of children)			
1	537	86.2	0.253
2	473	88.2	
3	376	89.9	
4+	737	89.4	
Occupation			
Farmer/other	1495	88.8	0.293
Business (petty vender, tailoring etc)	355	88.5	
Housekeeper/no job	215	84.7	
Civil servant	58	91.4	
Religion			
Islam	868	85.0	<*0.001*
Christian	1192	90.7	
Other	63	92.1	
Tribe			
Kurya	313	92.3	<*0.001*
Makonde	272	87.5	
Hangaza	206	92.2	
Zigua	204	92.2	
Zaramo	140	74.3	
Ndali	129	93.0	
Sukuma	120	83.3	
Others^a^	739	87.7	
Region (district)			
Arusha (Ngorongoro)	280	92.5	<*0.001*
Kagera (Ngara)	331	90.6	
Lindi (Lindi rural)	183	84.2	
Mara (Serengeti)	286	89.9	
Mbeya (Ileje)	188	93.1	
Mtwara (Newala)	193	88.6	
Mwanza (Ilemela)	235	89.4	
Pwani (Kisarawe)	190	72.6	
Tanga (Handeni)	237	89.9	

^a^ Sonjo, Mwela, Yao, Iraki, Chagga, Ha, Masai, Haya, Sambaa etc

### Questions regarding the anticipated malaria vaccines

**Fig. 2:**
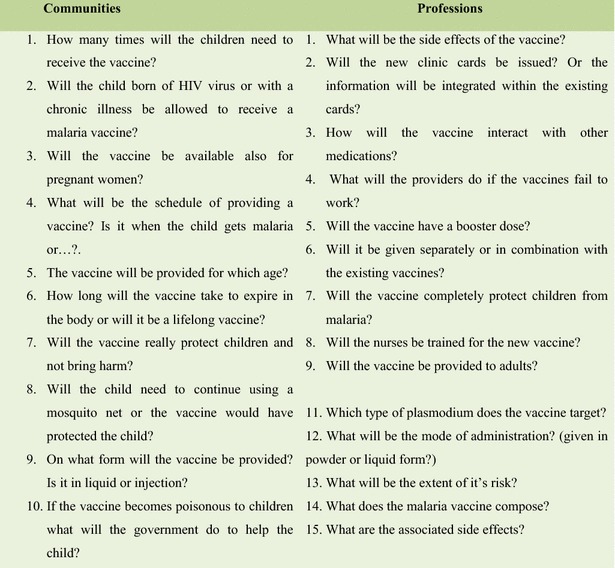
The common questions with regards to malaria vaccines by communities and professions

Despite a positive attitude towards the anticipated malaria vaccine, most participants had various questions with regards to the vaccine. However, there appeared to be similarities with regards to the questions asked by the communities and those asked by other professions. Most questions were mostly related to the side effects of the vaccine and the government response to them, efficacy, protective duration, composition, interaction with other medications, relation of vaccine schedule with existing EPI schedule, availability of the vaccine to the pregnant women, mode of administration (oral or injection?) and whether child born of HIV virus or with a chronic illness will be eligible for the vaccine? (Fig. 2).

## Discussion

The study findings suggest that stakeholders have a positive attitude towards the anticipated malaria vaccine and that their acceptance of the vaccine remains high despite the fact that it would be used parallel with other existing intervention strategies. Interestingly, the acceptance level also remains significant though the malaria vaccine is less likely to provide full protection. This outcome could be a reflection of how malaria is seriously perceived in the communities being studied. Furthermore, they may be willing to accept the new malaria interventions as long as they will (to some extent) contribute to the reduction of malaria, especially among children. Similarly, a study in Kenya also found that participants understood that malaria is a serious problem that no single tool can be used to combat it, which influences their acceptance of malaria vaccine [14]. Acceptance of malaria vaccine was also observed in studies conducted in Ghana [13] where the views of various professions and communities also reflected a positive opinion towards the introduction of malaria vaccine as a preventive tool. The study finding that stakeholders would still maintain the acceptance of malaria vaccine in the context of existing malaria intervention strategies is in line with the overall idea of introducing the vaccine which is not meant to replace the existing malaria interventions but rather to compliment it [11].

In this study, social cultural aspects emerged as factors associated with the acceptance of malaria vaccine. These factors include religion (Christians were more willing than Muslims to accept the vaccine), religion (Ndali tribe was less willing to accept the vaccine than the other tribes), and civil servants were more willing to accept the vaccine than the farmers. This finding corroborates with evidence from other countries in Africa [24] and elsewhere [25] where religion and ethnicity were found to influence health care utilization. Specific evidence also indicates that religion and ethnicity are associated with vaccine awareness and acceptance i.e measles vaccine and Human Papillomavirus (HPV) [25, 26].

The differences in vaccine acceptance based on religion, ethnicity and occupation as observed in this study could also reflect that people’s values, preferences and expectations would sometimes constrain their acceptance of a particular health care programme. These could originate from the culture in which the social interaction is taking place, which in turn govern their decisions about how they should pursue a recommended health intervention [27]. Although other studies have found that the quality of care i.e. congestion, delays, and the perceived attitudes of the health care providers [14], access to services, reliability of services fear of side effects, and parental beliefs and conflicting priorities [28] constraints immunization services, this study shows that in addition to individual and health system factors, the social cultural aspects may play a significant role in influencing the differential acceptance of vaccination programmes. This is central in this paper, and it lends support to the views of other researchers that people may not automatically use a health intervention once introduced [14], and in the context of a vaccine, if the known barriers are not addressed may lead to under-utilization of immunization coverage [16, 29].

Currently there is a strong recognition globally that health is socially determined and that social-structural aspects are responsible for health inequity. As found in this study, religion and ethnicity may play a significant role towards inequity in immunization coverage. Health inequity is known to be a set back to the wider health development, and this could be addressed by examining the wider social and structural aspects that increase vulnerability to diseases [30–33]. Evidence in Nigeria indicates that the community tailored interventions have proven to be effective in increasing the utilization of polio vaccination [34]. As such, the public health communication strategy that seek to promote the available immunization services as well as the anticipated malaria vaccine could be made effective if tailored within the broader social aspirations and cultural differences existing in the locally contextualized environment.

This study also found that the community and other professionals have multiple expectations and questions that relate to the anticipated malaria vaccine. It is important that the Tanzanian Immunization Department, malaria vaccine initiative, and other malaria stakeholders clarifies the questions and expectations prior to or parallel with the introduction of the malaria vaccine and provide the correct knowledge about the added value of malaria vaccine in lay man’s language to avoid any misconceptions about the anticipated malaria vaccine. The voices of communities and that of the health care professionals are important and should be considered for better informed decisions, policy recommendation, planning and designing of a communication strategy. Failure to account for community’s prior information that could enlighten policy makers on what is needed to be considered before the implementation of the intervention was found as one of the factors that could delay the public acceptance of the proposed intervention [14].

## Conclusions

Understanding stakeholders’ acceptability and perspectives regarding the anticipated malaria vaccine in the context of other ongoing malaria interventions is crucial for appropriate policy decisions in Tanzania. Stakeholders’ high acceptability of the anticipated malaria vaccine, even when it is less likely to provide full protection may reflect the extent to which malaria interventions, are needed in the study areas. However the questions raised by the communities reflect the need to clarify some misconceptions and provision of correct knowledge regarding the vaccine. The optimal acceptance and utilization of the anticipated malaria vaccine may require addressing of the social cultural aspects that could impede the utilization of the vaccine.

## Key messages and recommendations

The fact that the views of various community groups reflect a willingness to undertake malaria vaccine parallel with the existing malaria intervention strategies (such as ITNs) could be one of the strengths within the National Malaria Control Strategy in Tanzania. This aspect may need to be emphasized during the implementation phase of malaria vaccine, since the vaccine may not provide full protection. Communities are also ready to accept a partial efficacy malaria vaccine, which may be useful in guiding policy recommendations toward the vaccine in the country.

Although stakeholders possess a positive opinion towards the anticipated malaria vaccine there is much on which the Tanzanian Immunization Department, malaria vaccine initiative, and other malaria stakeholders, need to undertake for optimal acceptance and utilization of the vaccine. Based on the findings the following recommendations can be made:

- The communication strategy should clarify the questions and expectations raised by stakeholders prior to or parallel with the introduction of the malaria vaccine in lay man’s language to avoid any misconceptions about the anticipated malaria vaccine.
- Issues on religion, ethnicity, occupation and region should be considered for the designing of culturally based interventions to increase the acceptability and effectiveness of vaccine programmes.
